# Evaluation of physical exercises to assess weaknesses in physical abilities related to fall risk among adults in different ages

**DOI:** 10.1186/s13102-026-01604-0

**Published:** 2026-02-24

**Authors:** Mia Folke, Annica Kristoffersson

**Affiliations:** Department of Computer Science & Engineering, Mälardalen University, Västerås, Sweden

**Keywords:** Fall risk assessment, Physical ability, Working age, Early physical decline, Digital test, Feasibility study

## Abstract

**Background:**

Falls are the leading cause of injury for people of most ages, and the prevalence of falls increases with age. Fall risk is associated with the specific physical abilities balance, strength, and neuromuscular ability. This article describes a set of 12 physical exercises to assess these specific physical abilities related to fall risk.

**Method:**

A convenience sample of 50 participants (26 men — 20–72 years of age, 24 women — 21–73 years of age) were recruited to this feasibility study to evaluate whether the set of 12 physical exercises and combinations of them can be used as a test to identify early weaknesses and severity of weaknesses in specific physical abilities and to get an indication of whether the physical exercises can identify age-related differences in specific physical abilities. They performed the physical exercises barefoot on a non-slippery floor. A test leader ensured that the participants executed the physical exercises correctly and determined whether the participants passed or failed the physical exercises according to pre-set pass criteria.

**Results:**

Eleven of the physical exercises can assess weaknesses in specific physical abilities. In future tests, the physical exercise *Short jump* should be excluded from the set of physical exercises due to large variations in execution. The 11 participants younger than 30 years of age (8 men, 3 women) did not show any weaknesses. Nineteen of the 29 participants, 30–60 years of age (9 men, 10 women), and all 10 participants above 60 years of age (6 men, 4 women) showed weakness in at least one specific physical ability.

**Conclusions:**

Eleven out of the 12 physical exercises evaluated in this feasibility study and combinations of them can be used to identify weaknesses in specific physical abilities related to fall risk. Performance on physical exercises, alone or in combination, can also be used to determine the severity of the weaknesses in the three specific physical abilities. The results indicate that the physical exercises can identify age-related differences in specific physical abilities.

## Background

### Epidemiology

Falls are the leading cause of injury for people of almost all ages [1, 2] requiring a life-course approach to fall prevention [3]. In the report Step Safely, the World Health Organization (WHO) underscores the growing problem of falls due to ageing populations in combination with a more sedentary lifestyle [3]. Globally, approximately 33% of people over 65 years of age fall at least once a year according to WHO and the risk of falling is increasing with age [3]. Coordinated analyses of four population-based cohort studies from Australia, Ireland, the Netherlands, and Great Britain revealed that the prevalence of falls increases sharply between 40 and 64 years of age [4]. It is estimated that occupational falls resulted in 36,000 global deaths in 2017 [5]. Falls are the most common work incident in Sweden with approximately 11,000 severe falls annually [6]. Among these falls, approximately 8,000 are ground level falls. In Sweden, the rate of occupational falls on ground level among women is highest for nurses, care assistants, pre-school teachers, teachers, cleaners, and similar occupancies. Among men, the rate is highest among construction workers, professional drivers, industry workers, janitors and similar occupancies [6]. The proportion of adult workers who suffer from fall injuries increases with age and is greatest among older working women [6]. Among hospital employees in the US, slips, trips, and falls are the second most common cause of injury leading to lost workdays [7]. On a global level, data on occupational falls is not reported in a standardized way following the International Classification (ICD) codes available [3]. However, slippery, cluttered and unstable surfaces increase the fall risk both among workers and older adults [3]. Based on a global survey and literature review, the WHO report "Step Safely" [3] outlines physical activities allowing children to develop balance, strength, control and coordination as one of the preventive actions. Among preventive actions for both older workers and older adults are performing exercises challenging balance and increasing lower-limb strength [3]. To minimize the risk of falling in case of slipping or tripping, training strength, balance, reflexes and coordination is important.

### Physiological mechanisms

Muscle activity is based on inherent reflexes, and it is possible to modify these reflexes through targeted repetitive exercise [8]. When aging, the neuromuscular ability is affected. Aging is therefore affecting balance control [9], the ability to take a quick recovery step in the case of imbalance [10], and the peak torque, i.e., the ability to perform rapid ankle dorsiflexion and plantarflexion [11]. Balance control is complex and apart from neuromuscular ability, it involves also vision, the organ of balance (the vestibular system) in the inner ear, and muscle strength. Muscle strength decreases with age [9, 12]. As early as at 40–50 years of age, gait instability begins to increase at an accelerated rate [13]. Musculoskeletal conditions, and therefore physical ability, may be more related to fall risk than other fall risk factors for people 50–64 years of age than for older adults [14]. It has also been shown that 45–55-year-olds can have weaknesses in physical abilities related to fall risk without being aware of them [15].

The major burden of disabilities arises from age-related loss in moving by the age of 60 [16]. Decline in physical ability before the age of 50 is mainly due to a lack of physical exercise rather than biological aging [17]. While not defining middle-aged adults in terms of age, Brown and Covinsky [18] reported that middle-aged adults’ capacity to recover from functional impairment is high and found the middle-aged adults ideal for preventing or delaying functional impairments through physical exercise interventions. In a large cohort study, Blodgett et al. [19] investigated longitudinal associations between one-legged balance (at 53 and 60–64 years of age) and the number of self-reported falls within the last year (at 60–64 and 68 years of age) for more than 2,000 individuals. A better balance at 53 years of age was associated with a lower number of recurrent falls at 60–64 and 68 years of age. A similar association between balance at 60–64 years of age and recurrent falls at 68 years of age was found. Those able to balance only 15 s instead of 30 s at 53 and 60–64 years of age were at a greater risk of falling at 68 years of age [19].

Therefore, addressing weaknesses in specific physical abilities at an early age to prevent falls is highly important regardless of whether they are age-related or not.

### Literature gap

In 2017, the WHO [16] noted that methods for controlling and monitoring early age-related declines in physical ability related to increased fall risk are lacking. The WHO therefore considered it important to develop techniques/methods to identify these declines [16]. Brown and Covinsky [18] concluded that the development of improved prognostic tools to identify those at risk and the development of interventions to prevent or delay functional impairments are needed.

There are several physical tests and questionnaires for assessing fall risk. It has been found that several questionnaire-based fall assessment tools have limitations [20] and that there is no physical exercise in isolation which can accurately predict fall risk [21].

A systematic review of the association between performance during one-leg stands and fall risk for community-dwelling healthy older adults concluded that the one-leg stand test alone is not a useful tool for assessing fall risk in clinical settings [22]. In a 5-year population-based study with 273 women being 36–57 years of age at baseline, there was no correlation between fall risk or recurrent falls, and either of the measures leg muscle strength, Timed up and go (TUG) test, Step test, Functional reach test, and Lateral reach test [23]. These assessments, which are commonly performed by a professional therapist, were developed to identify an increased fall risk among older people who may already have fallen. In addition, some physical tests, i.e., the TUG test [24] include only one complex physical exercise. For example, a failed test may provide information that an individual has a balance problem or insufficient muscle strength without identifying which physical ability/abilities is/are declined. Another problem is that the tests for assessing fall risk generally do not assess all relevant physical abilities related to fall risk. Therefore, fall risk may remain undetected if the individual has weaknesses in other physical abilities than those assessed.

Existing tests to assess fall risk for older adults have limitations when it comes to assess fall risk and which specific physical abilities that are weakened. The assessment criteria are often set such that severe weakness in physical ability is needed to fail the tests. However, early weaknesses in physical ability/abilities related to fall risk increase the fall risk and if not being reversed, the risk of falling will become high. As WHO [16] mentioned, tests assessing early weaknesses in physical ability are of importance. This kind of test is lacking in literature. Thereby, there is also a lack of customized tests for working age adults who often have only early weaknesses. However, some researchers are working in the area and in a recent study [25], machine-learning on features obtained through video-based analysis of the first three steps was used to detect high fall risk among workers being 40–60 years of age walking 10 m. The severity of the fall risk was determined by self-reported falls during the past year. The fall prediction model used was more accurate for men than women.

### Study rationale

This feasibility study aimed (i) at evaluating whether a set of 12 physical exercises and combinations of them can be used to identify early weaknesses and severity of weaknesses in the specific physical abilities balance, strength and neuromuscular ability, and (ii) to get an indication of whether the physical exercises can identify age-related differences in specific physical abilities.

The first hypothesis was that the performance on each of the physical exercises and the performance on combinations of them can be used to identify weaknesses in each specific physical ability and the severity of these weaknesses.

The second hypothesis was that this set of physical exercises can be used to observe age-related differences in the specific physical abilities.

In our previous study [15], a number of physical exercises were used to identify early declines in physical ability. The physical exercises were evaluated with 36 participants being 45–55 years of age. We found that only the ability to take quick steps was limited for many of the participants. Therefore, the third hypothesis was that having limitations only regarding the ability to take quick steps would be common for participants in that age group also in this feasibility study.

Our vision is that the set of physical exercises can be used in a test for screening of early weaknesses in physical abilities related to fall risk already in the working age. To help individuals to perform the test without healthcare professionals, a digital version of the test is under development.

## Methods

A convenience sample of 50 participants (20–73 years of age, 26 men and 24 women) were recruited to this feasibility study which aimed to evaluate whether a set of 12 physical exercises and combinations of them can be used to identify early weaknesses and severity of weaknesses in specific physical abilities and to get an indication of whether the physical exercises can identify age-related differences in specific physical abilities. Eleven of these participants were younger than 30 years of age and 10 were older than 60 years of age. The recruitment of 50 participants was performed mainly through university news items on the web and social media, and through word-of-mouth. The inclusion criterion was being 20–74 years of age. The exclusion criteria were: essential functional disabilities (e.g., amputated, wheelchair bound, suffered from stroke, severe injuries in joints or muscles), or cognitively impaired. None of the participants who showed interest in the study was excluded. Therefore, they received an invitation letter with detailed information on the study and were asked to provide their informed consent prior to taking part in the measures. All participants completed all measures in the study.

The feasibility study was approved by the Swedish Ethical board, Diary number: 2022–06690-01.

The remainder of this section describes the experimental procedure, the physical exercises, and the data analysis.

### Experimental procedure

Prior to performing the physical exercises, the participants were asked to provide information on their age, biological gender (man or woman) and how many times they had fallen during the past six months. Those who reported one or several falls were asked to describe the situation/s.

Thereafter, they performed all physical exercises barefoot and on a non-slippery floor. The participants received information on how to execute each physical exercise and a test leader ensured that the participants understood how to execute the physical exercises correctly. If needed, the test leader provided correcting instructions such that all participants knew how to execute the physical exercise. The test leader also determined whether the participants passed or failed each of the physical exercises according to pre-set pass criteria in Table 1. The participants were told to make a few attempts at each physical exercise if not passing them directly to ensure that they did not make a mistake the first time. To make the participants feel safe and do their best on the physical exercises, the test leader ensured that the participants had a chair behind them during squats, a non-slippery mat. The test leader also stood close to them, ready to act if the participants lost balance. Therefore, all participants got the same conditions for performing the test.

**Table 1:** Short descriptions of the physical exercises included at each level, pass criteria, and the assessed physical abilities

Level	Physical exercise	Short description	Pass criteria	Assessed physical ability
1	(i) Feet together stand	Stand with feet together for 20 s	• Hands attached to the waist • Feet remaining in their positions	Balance
1	(ii) Tandem stand	Stand in tandem position for 20 s	• Hands attached to the waist • Feet remaining in their positions	Balance
2	(iii) One-leg stand	Stand on one leg for 60 s with the other foot touching this leg. Assessed per leg	• Hands attached to the waist • Feet remaining in their positions	Balance, Strength
2	(iv) Stand on toe tips	Stand on toe tips with hands as high as possible for 5 s	• Hands remaining up • Remaining on toe tips without adjusting position	Balance
2	(v) One-leg stand with head turn	Stand on one leg with the other foot touching this leg while turning head one time in each direction. Assessed per leg	• Hands attached to the waist • Feet remaining in their positions	Balance
2	(vi) Balance walk	Perform 10 steps heel to toe on straight line	• Hands attached to the waist • Putting the foot down with heel directly in front of the other foot’s toe • Not touching the ground at any other places than at the line	Balance
2	(vii) Put on socks	Put on a sock while standing on one leg. Assessed per leg	• Not using any support such as wall, table, etc • Remaining in position on the foot standing on • Keeping the foot above ground till sock is fully on	Balance
2	(viii) Stand up on one leg	Stand up from edge of chair one time on one leg and maintain balance for 5 s after standing. Assessed per leg	• Getting up to standing without leaning back with the upper body • Getting up to standing without using hands to push off	Strength
			• Only assessable if passing strength • Maintain balance on one foot for 5 s without the other foot touching the ground	Balance
2	(ix) Lunge	Perform one lunge. Assessed per leg	• Approximately 90° knee angle in lunge position for both legs without touching the ground with the knee • When returning with the foot, not touching the ground before being back in initial position	Strength
			• Only assessable if passing strength • Hands attached to the waist	Balance
2	(x) Short jump	Perform one 70 cm long jump and maintain balance for 5 s after landing	• Jumping 70 cm	Strength
			• Remain with the feet on their landing positions for 5 s after landing	Balance
2	(xi) Chair squats	Perform three *Chair squats* towards edge of chair at each speed: 50, 75, and 100 bpm	• Turning between ascending and descending directions according to metronome speed • Touching the edge of the chair for every squat	Strength, Neuromuscular ability
2	(xii) Side steps	Perform three *Side steps* (take two steps to the left and two to steps to the right) at each speed: 100, 150, 175, 200, 225, and 250 bpm	• Take steps according to metronome speed • Step length at least 40 cm	Neuromuscular ability

Both authors acted as test leaders. Five participants were assessed by both authors (20, 20, 24, 32, and 55 years of age). The first author assessed 36 participants alone (20–73 years of age), and the second author assessed the remaining nine participants alone (38–56 years of age). To ensure consistency in the assessment across all participants, both authors strictly assessed the performance according to the pre-set pass criteria in Table 1.

In this feasibility study, each participant performed all 12 physical exercises at one occasion together with a test leader.

### Physical exercises

The 12 physical exercises (Table 1) were selected to support the assessment of the specific physical abilities *balance*, *strength*, and *neuromuscular ability*. To reduce the fall risk while participating in the study, the physical exercises were divided into two levels. If a participant did not pass the two physical exercises at level 1 (Table 1), the participant should not perform the remaining ten physical exercises, i.e., level 2. To pass a physical exercise, the participant needed to fulfill all pass criteria for the assessed specific physical ability. As shown in Table 1, some physical exercises were used to assess more than one specific physical ability. For some of these, there were different pass criteria for each of the specific physical abilities.

*Static balance* was assessed by asking the participants to: (i) stand still with their feet close together (*Feet together stand*) for 20 s, (ii) stand still with their feet right in front of each other (*Tandem stand*) for 20 s, (iii) stand still on one leg with the other foot touching this leg (*One-leg stand*) for 60 s, and (iv) stand still on toe tips and try to reach something above the head (*Stand on toe tips*) for 5 s. The first three physical exercises were inspired by tests used for older people, the Four-Test Balance Scale [26].

*Dynamic balance* was assessed by asking the participants to: (v) stand still on one leg with the other foot touching this leg while simultaneously turning the head and looking to the side, to the other side and back to the starting position (*One-leg stand with head turn*), (vi) walk 10 short steps on a straight line, bringing the heel of the leading foot close to the toe of the other foot (*Balance walk*), (vii) stand still on one leg and put on one sock per foot without the use of any external support and without moving the foot standing on (*Put on socks*), (viii) sit on a chair and rise up using only one leg, i.e., without touching the floor with the other foot, using the upper body to gain momentum, or using the arms to pull or push (*Stand up on one leg*), (ix) take a step forward and perform a *Lunge* with both knees at 90 degrees at lunge position and with the back knee almost touching the ground, and thereafter return to the starting position without the front foot touching the floor before fully back, and (x) perform a 70 cm jump and maintain balance for 5 s after landing (*Short jump*).

The physical exercises (iii) *One-leg stand,* (viii) *Stand up on one leg*, (ix) *Lunge,* and (x) *Short jump* assessed both balance and strength. The latter physical exercise, (x) *Short jump*, also assesses if the participant has the peak torque required to be able to jump. Asking the participants to stand still on one leg for 60 s allowed for the assessment of strength of the muscles involved since a muscle’s endurance correlates with the load intensity of the muscle [27, 28].

Strength was further assessed by asking the participants to perform squats down to the edge of a chair (xi) *Chair squats*. The participants were asked to perform three *Chair squats* at 50 bpm and asked to repeat it at two progressively higher speeds. The speed levels 1–3 used in our previous study [15] were used also in this study and correspond to 50, 75, and 100 bpm. The physical exercise (xi) *Chair squats* is a modified version of the 30-s chair stand test [29] in which the number of times that a person can stand up from a chair within 30 s needs to be counted, as well as the 5-Repetition Chair rise [30] on time. Rather than letting our participants perform the chair stands for such a long time, we assessed their ability to perform squats quickly. This removed the need to count the number of squats and reduced the number of squats needed to be performed. This reduced the risk of tiring out the participants before performing the next physical exercise. Since (xi) *Chair squats* is performed at a high speed, it also assessed neuromuscular ability and peak torque for the knees.

To further assess neuromuscular ability, (xii) *Side steps* was used. The participants were asked to take a sideways step (step length at least 40 cm which was marked with tape) and put the other foot next to it. The participants should take two such steps to the left and two to such steps to the right, counted as one Side step. They should perform three Side steps at 100 bpm. The participants were asked to repeat it at five progressively higher speeds. The speed levels 1–6 used in our previous study [15] were used also in this study and correspond to 100, 150, 175, 200, 225, and 250 bpm. This physical exercise was inspired by the Four Square Step Test [31] in which individuals need to take quick steps over sticks laying on the ground to indicate four squares. The individuals need to take one step in each square as quickly as possible without changing the direction of the toes. This is associated with an increased fall risk. Therefore, we selected an exercise requiring directional changes without the risk of tripping on obstacles.

A metronome was used to help the participant maintain the right speed while performing (xi) *Chair squats* and (xii) *Side steps*. For both physical exercises, the movements should be performed in synchrony with the sound. If the participants were unable to follow only the sound, the test leader performed the physical exercise together with the participants. The speed was progressively increased in order to teach the participants how to perform the physical exercises at a lower speed before attempting to perform them at a higher speed. Safety was an additional reason for progressively increasing the speed. To avoid falls, the participants should not make attempts at higher speeds than they can handle. The speed at which the participants can perform the physical exercises provides an indication of the severity of weakness in the assessed physical abilities. However, to pass (xi) *Chair squats* and (xii) *Side steps*, participants should be able to perform them at the highest speeds.

During a pre-study, we observed that participants can step quick in one direction but struggle to keep the speed when changing direction. Here, the use of a metronome made it possible to detect this problem at a lower speed, a problem that could have been missed if the time for performing a specific number of (xii) *Side steps* had been measured. Thereby, the risk of falling while testing was reduced. The highest speed level used for Side steps was selected based on the pre-study showing that it is possible to perform (xii) *Side steps* at the speed after a training period for most people. The highest speed level used for (xi) *Chair squats* was selected since young participants in a pre-study could not “go down” (ascend) quicker during the (xi) *Chair squats.*

There are studies reporting on high test–retest reliability for several tests used as inspiration for the physical exercises in our test, 5-Repetition Chair Rise [32], Four Square Step Test [31], and One-leg stand [33]. Therefore, it is realistic that test–retest reliability would be high also for the physical exercises included in the test. This will be evaluated in future work.

### Changes in physical exercises in relation to previous study

The set of physical exercises and how to execute them differ from those in our previous study [15]. The physical exercises (v) *One-leg stand with head turn* and (vii) *Put on socks* were added to increase the possibility to identify a weakness in dynamic balance. To ensure a reliable assessment, the pass criteria outlined in Table 1 were used. The participants were required to perform the physical exercises at level 1, i.e., (i) *Feet together stand*, and (ii) *Tandem stand*, and level 2, i.e., (iii) *One-leg stand*, (v) *One-leg stand with head turn*, (vi) *Balance walk* and (ix) *Lunge* with their hands attached to the waist. If a participant struggled with maintaining balance such that the hands were not attached to the waist while performing the physical exercise, it was considered as a “failed” physical exercise. For each physical exercise assessing balance, the participants were instructed where to have their feet. If a foot was moved to an incorrect position, the physical exercise was considered as a “failed” physical exercise. This placement of feet and hands also made it possible to assess the participants’ use of stabilizing muscles instead of changing the center of gravity to maintain balance. The physical exercise *Semitandem stand* was replaced by the physical exercise (i) *Feet together stand* to ensure that the first physical exercise is easy to understand and perform for most participants.

In the previous study [15], two step combinations were used to test the ability to take quick steps, (xii) *Side steps* and a physical exercise called *Two steps forward and two steps back*. While testing in a pre-study, we observed that the step combination was associated with a risk of falling backwards. Therefore, the physical exercise was excluded from the test to minimize the risk of falling.

The physical exercise *Toe raises* was excluded since the participants in our previous study [15] were unable to assess the performance by themselves. Instead, the physical exercise (x) *Short jump* was added to assess strength and if the participant has the peak torque required to be able to jump and simultaneously assess balance while landing.

### Data analysis

The physical exercises passed or failed by the 50 participants were summarized. In addition, weaknesses in each specific physical ability were summarized for the 29 participants who failed at least one physical exercise and thereby had any weakness in their physical ability.

The participants’ specific physical abilities (balance, strength, and neuromuscular ability) were calculated by summarizing the performance on the physical exercises assessing each specific physical ability. For the physical exercises on which a participant could pass or fail, the result was set to 2 if passing with both legs, 1 if passing with one leg and 0 if failing with both legs. For the physical exercises (xi) *Chair squats* and (xii) *Side steps*, the highest speed at which the participants could perform them was used as the result. Here it should be noted that these speeds were converted into adjacent numbers instead of disjoint discrete numbers. The three speeds possible for (xi) *Chair squats* were converted to 1–3, and the six speeds possible for (xii) *Side steps* were converted to 1–6. Hence, the participants’ balance, strength, and neuromuscular ability were weighted summaries with maximum scores 11, 10 and 9 respectively.

After visualizing each participants’ balance, strength, and neuromuscular ability across age using scatter plots, the Spearman correlation rank coefficient *r*_*s*_ along with the corresponding *p-*values were calculated to identify any relationships between the specific physical abilities and age for three groups (all participants, all men, and all women).

In addition, despite the small sample size, an exploratory analysis of relationships between age and abilities was conducted. Mean scores, standard deviations and median scores of the specific physical abilities were calculated for three age groups. Then, the non-parametric Kruskal–Wallis Test was used to perform exploratory calculations of differences between the age groups. The analysis was performed for all participants, all men, and all women.

## Results

This section presents the results of the analysis of the performance on the physical exercises. In addition, information on falls reported by participants is provided.

### Participants’ performance on the physical exercises

All 50 participants passed the two physical exercises at level 1, i.e., (i) *Feet together stand* and (ii) *Tandem stand*. Hence, all 50 participants performed the ten physical exercises at level 2. Out of these ten physical exercises, all participants passed also (ix) *Lunge* and (x) *Short jump*.

There were 21 participants who passed all physical exercises. Eleven of those, being younger than 30 years of age (8 men —20, 20, 21, 23, 24, 28, 28, and 29 years of age, 3 women — 21, 22, and 23 years of age), and ten being 36–60 years of age (3 men — 41, 56, and 60 years of age, 7 women — 36, 43, 46, 53, 56, 56, and 57 years of age). In this study sample, none of the participants being older than 60 years of age passed all physical exercises.

Table 2 provides information on the performance of the 29 participants who failed at least one physical exercise. Only the eight physical exercises where at least one participant failed are included. For the participants who were unable to perform (xi) *Chair squats* or (xii) *Side steps* at the maximum speed, information on the highest speed at which they were able to perform them is provided.

**Table 2:** Age, gender, and performance for the 29 participants who failed at least one physical exercise

Demographics			Balance, Strength	Balance				Strength	Balance	Strength, Neuromuscular ability	Neuromuscular ability
Age	Gender	Fall history past six months	(iii) One-leg stand	(iv) Stand on toe tips	(v) One-leg stand with head turn	(vi) Balance walk	(vii) Put on socks	(viii) Stand up on one leg	(viii) Stand up on one leg	(xi) Chair squats	(xii) Side steps
32	M										225
33	F										225
37	M										175
38	F										225
39	F										200
39	M		L	x	RL	x		RL		50	150
40	M							L			175
47	M				L			L			
48	M									75	175
49	F		R		R			RL		75	175
49	M			x	RL		x	RL			200
51	F										200
51	M										175
51	F										150
53	F				R		x				200
54	F	1	RL					RL		75	225
55	F		RL		RL			L			
57	M	1									200
58	F	1									200
62	F	8									225
63	M		RL								175
66	M		RL								200
66	M		L		RL		x				175
67	M		R		RL			RL			175
69	F		R						R		200
70	M		RL						L		225
72	M	1	RL		RL				RL		175
73	F		RL								175
73	F		RL		RL		x	RL		75	150

Failed physical exercises are indicated by an x when the physical exercise is involving both legs at the same time, with R (right leg) and L (left leg) when assessing each leg separately, or with a number. The numbers indicate the highest speed at which the physical exercise could be performed. An empty box means that the participants passed the physical exercise. Male (M), female (F)

Among the 29 participants who failed at least one physical exercise, 11 participants (32–62 years of age) failed only (xii) *Side steps* which was used to assess neuromuscular ability. Only two out of these 29 participants passed this physical exercise. Hence, the physical exercise indicating a weakness in physical abilities related to fall risk for the largest number of participants in our sample of participants was (xii) *Side steps*.

The physical exercises (iii) *One-leg stand,* (iv) *Stand on toe tips,* (v) *One-leg stand with head turn,* (vi) *Balance walk,* and (viii) *Put on socks* were used to assess balance. Eight participants failed (iii) *One-leg stand* with both legs and five failed it with one of their legs. Of the 13 participants failing (iii) *One-leg stand* with at least one leg, all participants younger than 60 years of age also failed other physical exercises assessing strength. For those older than 60 years of age, 9/10 participants failed (iii) *One-leg stand*. Only two of them failed any other physical exercise assessing strength.

Seven participants failed (v) *One-leg stand with head turn* with both legs and three failed it with one of their legs. One participant failed (vi) *Balance walk*, two participants failed (iv) *Stand on toe tips,* and four participants failed (viii) *Put on socks*.

The physical exercise (viii) *Stand up on one leg* was used to assess both strength and balance. If a participant was not strong enough to stand up on one leg, balance could not be assessed. Six participants were not strong enough to stand up on either leg, and six were strong enough to stand up with one of their legs. Three of the six participants who were able to stand up with one of their legs were also able to maintain balance on that leg. Another three participants were strong enough to stand up on each leg but unable to maintain balance. One of them struggled with maintaining balance on both legs, and the other two struggled on one of the legs.

**Fig. 1:**
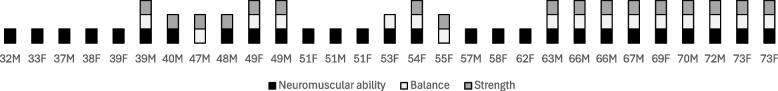
Age, gender (M/F), and weakened specific physical abilities for the 29 participants having weakness

A summary of weaknesses in specific physical abilities for the 29 participants who failed at least one physical exercise is presented in Fig. 1. All but two of these participants had a weakness in their neuromuscular ability. Weakness in neuromuscular ability was found among participants in the age range 32–73 years of age. Twelve participants had weaknesses in both balance and strength. Six of these were in the age range 39–55 years of age. Hence, the youngest participant that had weaknesses in both balance and strength was 39 years of age. Two participants (40 and 48 years of age) had a weakness in strength but not in balance, while none of the participants older than 48 years of age had. One 53-year-old woman had a weakness in balance but not in strength.

For completeness, Table 3 provides a comparison of the specific physical ability scores across the three age groups < 30, 30–60, and > 60 years of age.

**Table 3:** Statistical summary of results per age group and specific physical ability

Specific physical ability	Age group	n	Mean ± SD	Median
Balance	< 30	11	11.00 ± 0.00	11
	30–60	29	9.93 ± 2.02	11
	> 60	10	7.70 ± 2.05	8.5
Strength	< 30	11	10.00 ± 0.00	10
	30–60	29	8.90 ± 2.02	10
	> 60	10	8.70 ± 2.15	10
Neuromuscular ability	< 30	11	9.00 ± 0.00	9
	30–60	29	7.48 ± 1.61	8
	> 60	10	6.40 ± 0.00	6

### Relationships between the specific physical abilities and age

As shown in Table 4, there was a moderate negative correlation between the specific physical ability balance and age for the two groups all participants and men. For women, there was only a weak negative correlation regarding balance. For men, there was also a moderate negative correlation between neuromuscular ability and age. For all participants and women, there was only a weak negative correlation regarding neuromuscular ability. There was a weak or very weak to non-existent negative correlation between age and strength for all three groups. Hence, there were no strong monotonic relationships between the specific physical abilities and age.

**Table 4:** Spearman correlation rank coefficient *r*_*s*_ between age and each of the specific physical abilities

Specific physical ability	*r*_*s*_ all	*r*_*s*_ men	*r*_*s*_ women
Balance	−0.53^**^	−0.61^**^	−0.47^**^
Strength	−0.25	−0.32	−0.24
Neuromuscular ability	−0.47^**^	−0.54^**^	−0.39

A correlation from 0 to 0.29 was considered as very weak or non-existent, a correlation from 0.3 to 0.49 as weak, a correlation from 0.5 to 0.69 as moderate, a correlation from 0.7 to 0.89 as strong and a correlation from 0.9 to 1.0 as very strong. * *p* < 0.05, ** *p* < 0.01

The exploratory calculations using the non-parametric Kruskal–Wallis test indicate that there are statistically significant differences in balance (H = 13.096, *p* = 0.001) and neuromuscular ability (H = 16.291, *p* = 0.000) when comparing the three age groups (< 30, 30–60, and > 30 years of age). Also for men, the results for this study sample indicate statistically significant differences in balance (H = 9.927, *p* = 0.007) and neuromuscular ability (H = 10.497, *p* = 0.005). For women, the results indicate only a statistically significant difference in neuromuscular ability (H = 1300.449, *p* = 0.000) but not balance (H = 3.070, *p* = 0.215) or strength (H = 0.455, *p* = 0.797). However, the p-values are exploratory and underpowered, i.e., not psychometrically validated.

### Reported falls

Eight participants in total (i.e., three who passed all physical exercises and five who failed at least one physical exercise) reported that they had fallen at least once during the past six months. Among the three participants who passed all physical exercises, a 20-year-old-man fell running up the stairs, a 24-year-old man fell 2–3 times on a floor slippery due to oil, and a 43-year-old woman fell on icy bare rock.

Among the five participants who failed at least one physical exercise, four reported one fall during the past six months. A 57-year-old man fell on a downward slope when the gravel on the ground had gone away. A 72-year-old man fell due to a bag in a bike wheel. A 54-year-old woman fell when walking in the forest, and a 58-year-old woman fell when running in the forest. A 62-year-old woman reported 8 falls. All these falls took place in the forest while running.

## Discussion

This study focuses on a set of physical exercises feasibility to assess early weakness and severity of weakness in specific physical abilities related to fall risk and if the physical exercises can identify age-related differences in specific physical abilities.

The results in this study confirm the first hypothesis, i.e., that the performance on each of the physical exercises and the performance on combinations of them can be used to identify weaknesses in each specific physical ability and the severity of these weaknesses. Some participants failing (iii) *One-leg stand* passed all other physical exercises assessing balance indicating that the participants have weakness in strength. This theory is also supported by knowledge that a weaker muscle has a shorter endurance time [27, 28]. Some participants failed the physical exercise (xi) *Chair squats*. One of them passed all other physical exercises except (xii) *Side steps*. This indicates that the reason for the failure on the physical exercise (xi) *Chair squats* was weakness in neuromuscular ability rather than weakness in strength.

The set of physical exercises provides the opportunity to assess the severity of weaknesses in each specific physical ability. None of the 50 participants failed the physical exercises (vi) *Balance walk* or (vii) *Put on socks* without failing any other physical exercise assessing balance. These physical exercises seem to assess more severe weaknesses in balance than other physical exercises. Also for strength, there are physical exercises like (ix) *Lunge* that allows for assessing more severe weaknesses. To capture the severity of the weakness in neuromuscular ability, the physical exercises (xi) Chair squats and (xii) Side steps were assessed with several speed levels rather than just pass or fail. To measure the time during which an individual is capable to stand in the one-leg position would capture the nuances in performance on one-leg stand. It should be noted that there are interactions between the specific physical abilities balance, strength, and neuromuscular ability in all physical movements a human performs. A severe weakness in one of them might influence the performance on a physical exercise assessing another specific physical ability and result in a failed physical exercise.

The results also confirm the second hypothesis, i.e., that this set of physical exercises can be used to observe differences in the specific physical abilities when aging. Decline in physical ability before the age of 50 is mainly due to a lack of physical exercising rather than biological aging [17]. Therefore, weaknesses observed for the participants younger than 50 years of age in this study are probably due to a lack of adequate physical training or injuries. With this set of physical exercises, which requires a higher level of physical ability to pass than the requirements in commonly used fall risk assessment tools, it is possible to detect early weaknesses in balance which are not related to strength and probably age-related. For example, one 53-year-old-woman had weakness in balance but not in strength. Among the younger participants (20–29 years of age) in this convenience sample, no weaknesses in physical abilities were identified. Even though there were only 11 participants being 20–29 years of age, the sample included both well-trained participants and those who had some difficulties when performing the physical exercises despite passing them all. These findings indicate that most healthy young people can perform all 12 physical exercises. Already at the age slightly above 30 years of age, there were participants in this sample who failed at least one physical exercise. Among the 29 participants being 30–60 years of age, 19 had a weakness in at least one specific physical ability. All except one of the participants being older than 60 years of age had weaknesses in at least two specific physical abilities. We can also observe that 9/10 participants older than 60 years of age failed (iii) *One-leg stand* without failing any other physical exercise assessing strength. Since this observation cannot be made for any of the participants younger than 60 years of age, this indicates that the set of physical exercises can identify weakness in balance for older adults that still have quite a good muscle strength. We see moderate negative correlations between results and age for the specific physical ability balance for all participants and all men in this sample. We also see a moderate negative correlation between results and age for neuromuscular ability for all men. Strong monotonic relationships between the results and age cannot be expected. The results indicate that independently of age, neuromuscular ability is the first weakness that will occur. The age of an individual is not relevant since weaknesses occur at different ages for different individuals. The convenience sample recruited to this study likely influenced the results. Among our participants, there were more women than men being 30–60 years of age and more men than women being older than 60 years of age. Power calculations were not relevant for this feasibility study. The exploratory calculations using the Kruskal–Wallis test with this study sample yielded statistically significant differences were found for balance and neuromuscular ability when comparing the three age groups for all participants and all men. For women, only the neuromuscular ability differed which may be due to the low number of women being older than 60 years of age and a high number of women being 30–60 years of age in the study sample. It is known that older female workers have a higher fall risk than men [6]. It is possible that some of the adults volunteering to participate in the study start feeling that they have weaknesses related to fall risk, which may have resulted in recruiting more women 30–60 year of age and more men older than 60 years of age.

In our previous study [15], all 16 participants who failed any physical exercise also failed at least one of the two physical exercises involving quick steps. Eleven of the 29 participants that failed at least one physical exercise in this study failed only the physical exercise (xii) *Side steps*. Thereby, also the third hypothesis was confirmed, i.e., that having a limitation regarding only the ability to take quick steps would be common in the age group 45–55 years of age also in this study. However, the age span was 32–62 years of age for the 11 participants failing only (xii) *Side steps* in this study. A speculation is that the physical exercise (xii) *Side steps* might be the physical exercise that detects the first sign of weakness related to fall risk. It also indicates that having a weakness causing limited speed on (xii) *Side steps* is not age-related*.* Rather, it seems that (xii) *Side steps* can identify early weaknesses that often occur before balance problems. However, further studies are needed to confirm this. In this study, we have used (xii) *Side steps* to assess the ability to take quick steps. It is possible to use other step combinations, for example the Four Square Step Test as long as it is possible to perform the physical exercise in a safe way and as long as the physical exercise is executed at a high speed. However, our aim is to develop a test for non-healthcare professionals. Therefore, we will continue to use (xii) *Side steps* to reduce the risk of falling.

Two participants (one 47-year-old man and one 55-year-old woman) passed (xii) *Side steps* without having failed other physical exercises. While performing the physical exercises with the test leader, one of them reported having several herniated discs in the back, and both reported that they had knee injuries. Injures might cause problems with balance or strength but as long as the individual can perform rapid steps, they may still pass (xii) *Side steps*.

The result from this study supports the choice of physical exercises included in the test. None of the 50 participants failed on the four physical exercises (i) *Feet together stand*, (ii) *Tandem stand*, (ix) *Lunge,* and (x) *Short jump*. It should be noted that (i) *Feet together stand* and (ii) *Tandem stand* are used as a safety level, i.e. the physical exercises at Level 1. A participant who fails any of these two physical exercises should not perform the other ten physical exercises. Therefore, (i) *Feet together stand* and (ii) *Tandem stand* cannot be excluded from the test. In our previous study [15], one participant failed (ix) *Lunge*. Therefore, it is important to keep (ix) *Lunge*. In this study, we observed that the participants performed (x) *Short jump* in varying ways. For example, some participants executed (x) *Short jump* as a real jump, whereas others more or less took a large step with just a small jump. Thereby, (x) *Short jump* assesses some of the participants’ ability to jump. For other participants, the pre-set pass criteria were not applicable since they did not execute it correctly. The physical exercise (x) *Short jump* assesses strength and balance which are assessed also in several other test exercises, and peak torque which is also assessed in (xi) *Chair squats*. The fact that none of the participants failed (x) *Short jump* shows that the physical exercise cannot be used to identify early weaknesses in specific physical abilities related to fall risk. Therefore, *Short jump* should be excluded from the set of physical exercises used in the test.

Since we saw while conducting the study that taking quick steps can be a fall risk, we determine that an individual should not perform (xii) *Side steps* unless having passed all other physical exercises. We therefore suggest that the test, and consequently the physical exercises, should be divided into three levels instead of only the two levels used in this study. Safety during the test was ensured by asking the participants to perform the physical exercises indoors on a non-slippery floor to minimize the risk of slippering.

Providing the participants with instructions on where to keep their feet and hands in fixed positions during most physical exercises assessing balance made it impossible for them to mitigate imbalance via larger body movements. In our previous study, as well as when healthcare professionals assess the patients’ ability to perform the physical exercise one-leg stand, the person is allowed to compensate for imbalance by changing the center of gravity by moving the hands or any of the legs. Our pre-set pass criteria enabled us to assess the participants’ ability to make small corrections to maintain balance.

The novelty of this study is the use of a set of physical exercises that can be used with or without help from healthcare professionals. These physical exercises can be used to (i) assess what specific physical abilities that are weakened, (ii) assess weaknesses in balance that seem to be age-related since some participants had a weakness in balance but not in strength, and (iii) assess early weaknesses since the physical exercises evaluated require a higher level of physical ability to pass than the requirements in commonly used fall risk assessment tools.

Three participants who passed all physical exercises and five participants who failed at least one physical exercise reported that they had fallen at least once during the past six months. Out of these eight participants, six reported one fall. By adding a question about the situation of the fall, information on the reason for falling was collected. The results reveal a limitation in the way fall history is often reported in research today. Our results suggest that some falls might be accidents not related to weaknesses in physical abilities. For our sample, it cannot be concluded that an individual has a high fall risk due to their self-reported fall risk history since some of them had no weaknesses in the specific physical abilities. However, one participant reported eight falls. All these falls occurred while running in the forest. This type of recurrent fall indicates a weakness in physical ability related to fall risk. This participant failed only the physical exercise (xii) *Side steps*. Individuals that have recurrent falls are defined as having a high risk of falling [34]. A speculation is that this physical exercise can be used to identify an increased fall risk. However, whether fall risk can be assessed with the physical exercises or not is not evaluated in this study. To confirm predictive validity, a study with a large sample size, and a prospective follow-up of falls over a period of 12 months or more is recommended. It should be remembered that this study indicates that just counting the number of falls can be misleading.

In our previous study [15], the participants being 45–55 years of age were not aware of their weaknesses in physical abilities related to fall risk. Given this fact, it is important to assess weaknesses in physical abilities for everyone already during working age. A test like this must also be possible to implement in real-world contexts. Here, several tasks need to be handled. Firstly, the physical exercises must be further evaluated for test–retest reliability. Secondly, the number of attempts allowed for each physical exercise must be stated. In this study, we allowed the participants to make a few attempts for each physical exercise. If we considered that a participant had almost passed a physical exercise but wanted to give up and go to the next physical exercise, we encouraged them to try one more time. If participants had tried up to five times without any chance to pass, we assessed it as failed and told the participant to stop trying. Thirdly, objective and clear pass criteria are needed if individuals are to be able to assess weaknesses in specific physical abilities with or without support from healthcare professionals. Some users may be able to perform the physical exercises and make the assessments by themselves while others may need assistance from another person while performing the test. Inter-rater reliability in the assessment of the pass criteria needs to be further evaluated.

Access to such a test could reduce the number of falls among adults of all ages and consequently fall-related injuries resulting in personal suffering. Apart from reducing the injuries, the economic burden related to for example sick leave for the individual, care costs for the society and various costs for the employer. If weaknesses in physical abilities related to fall risk are identified early, they can be reversed through directed physical training. This minimizes the risk of more severe weaknesses which is associated with a higher risk of falling [3, 34]. The improved physical ability resulting from the performance of directed physical training impacts the quality of life [3].

As mentioned in the background, a digital version of the test is under development. Even though digital protocols for other fall risk assessment tools have recently been evaluated with good results regarding feasibility of virtual administration of the TUG, 5-Repetition Chair Rise, and 1-min Sit-to-Stand tests [32], it does not mean that it is be possible to develop instructions such that non healthcare professionals are able to use the test on their own. Some kind of education or support when performing the test might be needed.

### Limitation

This feasibility study aimed at evaluating whether a set of 12 physical exercises and combinations of them can be used to identify early weaknesses and severity of weaknesses in the specific physical abilities balance, strength and neuromuscular ability, and to get an indication of whether physical abilities differ across ages.

The 50 participants performed the test at one single occasion. This makes it possible to evaluate the set of physical exercises and if they can be used to identify early weaknesses and the severity of weaknesses in the specific physical abilities balance, strength and neuromuscular ability. A limitation in the evaluation of the physical exercises performed is that test–retest reliability was not evaluated. However, the way in which users are instructed how to perform the physical exercises, the pre-set assessment criteria to determine whether users pass or fail, and the way in which the users are supported when assessing their performance on the physical exercises in a future test, regardless of if being performed in person or digitally, will affect the test–retest reliability. This feasibility study was conducted while still being in the design phase. Therefore, we decided to wait with test–retest reliability.

In this study, we calculated each participant’s specific physical ability by giving one point per passed leg and by giving points depending on highest speed passed for (xi) *Chair squats* and (xii) *Side steps*. Some physical exercises are significantly more difficult to pass than others. The unweighted summation score assumes an arbitrary equivalence between the difficulty of static tasks and speed levels. This scoring metric has not yet been validated for internal consistency.

The convenience sample of 50 participants was limited for getting an indication of whether the physical exercises can identify age-related differences in specific physical abilities. As discussed, strong monotonic relationships between the results and age cannot be expected. The limited sample made it impossible to perform any other statistical analysis. While the sample can provide an indication about weakness in specific physical abilities, it is important to acknowledge that the results are not representative for the whole population at these ages. The result might be biased due to the use of a convenience sample.

## Conclusion

In this feasibility study with a convenience sample of 50 participants, we found that combinations of a set of physical exercises can be used to identify early weaknesses in physical abilities related to fall risk. Performance on physical exercises, alone or in combination, can also be used to determine the severity of the weaknesses in different specific physical abilities.

The results give an indication of how physical abilities differ across ages. In this study sample, we identified weaknesses in neuromuscular ability already in a participant slightly older than 30 years of age. Weakness in only the neuromuscular ability was common among the participants being 30–60 years of age.

Screening adults using a test capable of identifying early weaknesses in the specific physical abilities balance, strength and neuromuscular ability could reduce the number of falls among adults of all ages and consequently fall-related injuries resulting in personal suffering and various costs. Weaknesses which are identified early can be reversed through directed physical training. Thereby, individuals can minimize their risk of more severe weaknesses and lower their fall risk.

## Data Availability

All data generated or analyzed during this study are included in this published article.

## Abbreviations

WHO: World Health Organization
TUG: Timed up and go
M: Male
F: Female
